# The *w*Mel Strain of *Wolbachia* Reduces Transmission of Chikungunya Virus in *Aedes aegypti*

**DOI:** 10.1371/journal.pntd.0004677

**Published:** 2016-04-28

**Authors:** Matthew T. Aliota, Emma C. Walker, Alexander Uribe Yepes, Ivan Dario Velez, Bruce M. Christensen, Jorge E. Osorio

**Affiliations:** 1 Department of Pathobiological Sciences, University of Wisconsin-Madison, Madison, Wisconsin, United States of America; 2 Programa de Estudio y Control de Enfermedades Tropicales (PECET), Universidad de Antioquia, Medellin, Colombia; The Connecticut Agricultural Experiment Station, UNITED STATES

## Abstract

**Background:**

New approaches to preventing chikungunya virus (CHIKV) are needed because current methods are limited to controlling mosquito populations, and they have not prevented the invasion of this virus into new locales, nor have they been sufficient to control the virus upon arrival. A promising candidate for arbovirus control and prevention relies on the introduction of the intracellular bacterium *Wolbachia* into *Aedes aegypti* mosquitoes. This primarily has been proposed as a tool to control dengue virus (DENV) transmission; however, evidence suggests *Wolbachia* infections confer protection for *Ae*. *aegypti* against CHIKV. Although this approach holds much promise for limiting virus transmission, at present our understanding of the ability of CHIKV to infect, disseminate, and be transmitted by *w*Mel-infected *Ae*. *aegypti* currently being used at *Wolbachia* release sites is limited.

**Methodology/Principal Findings:**

Using *Ae*. *aegypti* infected with the *w*Mel strain of *Wolbachia* that are being released in Medellin, Colombia, we report that these mosquitoes have reduced vector competence for CHIKV, even with extremely high viral titers in the bloodmeal. In addition, we examined the dynamics of CHIKV infection over the course of four to seven days post feeding. *Wolbachia*-infected mosquitoes remained non-infective over the duration of seven days, i.e., no infectious virus was detected in the saliva when exposed to bloodmeals of moderate viremia, but CHIKV-exposed, wild type mosquitoes did have viral loads in the saliva consistent with what has been reported elsewhere. Finally, the presence of *w*Mel infection had no impact on the lifespan of mosquitoes as compared to wild type mosquitoes following CHIKV infection.

**Conclusions/Significance:**

These results could have an impact on vector control strategies in areas where *Ae*. *aegypti* are transmitting both DENV and CHIKV; i.e., they argue for further exploration, both in the laboratory and the field, on the feasibility of expanding this technology beyond DENV.

## Author Summary

New approaches to preventing chikungunya virus (CHIKV) infection are needed because the endemic range of this virus is expanding and because current methods are limited to controlling mosquito populations, and this approach has not effectively controlled this virus. A promising candidate for arbovirus control and prevention relies on the introduction of the intracellular bacterium *Wolbachia* into *Aedes aegypti* mosquitoes. *Wolbachia* biocontrol has advanced from laboratory experiments demonstrating that *Wolbachia* reduces virus replication to small-scale field trials demonstrating that *Wolbachia* are capable of spreading through wild *Ae*. *aegypti* populations. This primarily has been proposed as a tool to control dengue virus (DENV) transmission; however, *Wolbachia* infections confer protection for their insect hosts against a range of pathogens including CHIKV in *Ae*. *aegypti*. Medium-scale *Wolbachia* deployments are imminent or in certain instances have commenced. Therefore, assessing whether or not *Wolbachia*-infected *Ae*. *aegypti* are effective against CHIKV will help inform the viability of *Wolbachia* biocontrol for CHIKV control. Our study provides valuable evidence that could justify expanding this type of control program to other *Ae*. *aegypti*-transmitted arboviruses, primarily CHIKV.

## Introduction

*Chikungunya virus* (CHIKV; *Togaviridae*, *Alphavirus*) has recently re-emerged out of Africa and caused explosive outbreaks of arthritic disease in Southeast Asia, India, Europe and currently the Americas [1–4]. The current outbreak in the Americas is cause for great concern because CHIKV is spreading nearly uncontrolled with at least 44 countries experiencing autochthonous spread [5]. Infection with CHIKV results in a severe febrile illness, called chikungunya fever. Clinically, it resembles dengue fever and several other arboviral diseases [6], but it is more associated with joint pain, which in some patients can progress to chronic arthralgia that lasts for months to years [7]. CHIKV disease can be highly debilitating and has a pronounced economic impact on both the affected individual and the countries which experience the outbreaks, resulting in great losses in productivity [8–10]. CHIKV is transmitted to humans by the mosquitoes *Aedes aegypti* and *Aedes albopictus*. The distribution of these mosquitoes explains the recent global spread of the virus and invasion of the Americas [4,5,11]. Both mosquito species have demonstrated the capacity to sustain CHIKV transmission cycles and both have been associated with CHIKV outbreaks [1]; however, the etiologic strain of CHIKV, a member of the old Asian lineage [12], causing the current outbreak does not efficiently infect *Ae*. *albopictus*, suggesting that most CHIKV transmission in the Americas will occur via *Ae*. *aegypti* [5].

Despite the continued spread of the virus, there remains no effective antiviral therapy or licensed vaccines. Therefore, new approaches to preventing CHIKV are needed because the endemic range of this virus is expanding and because current methods are limited to controlling mosquito populations. To date, mosquito control has not prevented invasion of this virus into new locales or controlled the virus when it arrives [13]. A promising candidate for arbovirus control and prevention relies on the introduction of the intracellular bacterium *Wolbachia* into *Ae*. *aegypti* mosquitoes. *Wolbachia* biocontrol has advanced from laboratory experiments demonstrating that certain strains of *Wolbachia* shorten the lifespan of the mosquito [14] while simultaneously reducing virus replication [15] to small-scale field trials demonstrating that *Wolbachia* are capable of spreading through wild *Ae*. *aegypti* populations [16–18]. This primarily has been proposed as a tool to control dengue virus (DENV) transmission [19–21]; however, *Wolbachia* infections confer protection for their insect hosts against a range of pathogens including for *Ae*. *aegypti* against CHIKV [22,23] and for *Ae*. *albopictus* against CHIKV [24]. As a result, this technology currently is being evaluated in five countries around the globe (Australia, Brazil, Colombia, Indonesia, and Vietnam) for its potential to control DENV transmission.

The approach is well-established that *Wolbachia* infection confers protection against DENV transmission by *Ae*. *aegypti*. In contrast, the ability of CHIKV to infect, disseminate, and be transmitted by *w*Mel-infected *Ae*. *aegypti* is far less established [23]. For example, van den Hurk et al. (2012) tested the *w*Mel strain of Wolbachia, but they only assayed *Ae*. *aegypti* vector competence for CHIKV at a single time point (12 days post feeding) with a single bloodmeal titer, and only could detect virus in the saliva via qRT-PCR [23]. And Moreira et al. (2009) tested the *w*MelPop strain of *Wolbachia* against CHIKV [22], which no longer is being utilized by the Eliminate Dengue Program (EDP) because mosquitoes infected with this strain of *Wolbachia* displayed reduced fitness in small-scale field releases [18]. Therefore, we assessed vector competence for CHIKV in *w*Mel-infected and *w*Mel-free *Ae*. *aegypti* from Medellin, Colombia, because at present our understanding of the ability of CHIKV to infect, disseminate, and be transmitted by *w*Mel-infected *Ae*. *aegypti* currently being used at *Wolbachia* release sites is limited. This becomes particularly important if one considers that vector competence of *Ae*. *aegypti* for certain viruses likely is governed to a large extent by vector genotype x virus genotype (G x G) interactions in genetically diverse, natural *Ae*. *aegypti* populations [25]. This challenges the general relevance of conclusions from laboratory systems that consist of a single combination of mosquito and virus genotypes [25,26]. These *Wolbachia-*infected mosquitoes were created as part of a collaboration with the EDP in Colombia and in the spring of last year (2015), medium-scale deployments of these mosquitoes began in the DENV metropolitan area of Medellin [see www.eliminatedengue.com/colombia]. Our results suggest that *Wolbachia* effectively blocks the transmission potential of Colombian *Ae*. *aegypti* for CHIKV and *w*Mel infection has no impact on the lifespan of mosquitoes as compared to wild type mosquitoes following CHIKV infection. To our knowledge, this is the first description of the effects of naturally acquired CHIKV infection (i.e., exposure to virus was accomplished by feeding on a viremic host) on *Wolbachia*-infected mosquito vector competence. All previous studies (including those mentioned for CHIKV, as well as the numerous studies described with DENV) have relied on animal blood spiked with cultured virus or have relied on viremic human blood from a membrane feeder. These data argue for the expansion of this technology to CHIKV in South America and are useful and germane in the broader context of CHIKV-mosquito interactions. Additionally, knowledge about factors shaping vectorial capacity (e.g., probability of daily survival) will be informative for a more accurate appraisal of CHIKV transmission and the likelihood of establishing *Wolbachia* infection in natural mosquito populations.

## Methods

### Ethics statement

This study was carried out in strict accordance with recommendations set forth in the National Institutes of Health *Guide for the Care and Use of Laboratory Animals*. All animals and animal facilities were under the control of the School of Veterinary Medicine with oversight from the University of Wisconsin Research Animal Resource Center. The protocol was approved by the University of Wisconsin Animal Care and Use Committee (Approval #V01380).

### Cells and viruses

African Green Monkey kidney cells (Vero; ATCC #CCL-81) were grown in Dulbecco’s modified Eagle medium (DMEM) supplemented with 10% fetal bovine serum (FBS; Hyclone, Logan, UT), 2 mM L-glutamine, 1.5 g/l sodium bicarbonate, 100 U/ml of penicillin, 100 μg/ml of streptomycin, and incubated at 37°C in 5% CO_2_. *Aedes albopictus* mosquito cells, (C6/36; ATCC #CRL-1660) were maintained in MEM supplemented with 10% FBS, 2 mM L-glutamine, 1.5 g/l sodium bicarbonate, 0.1 mM non-essential amino acids, 100 U/ml of penicillin, 100μg/ml of streptomycin, and incubated at 28°C in 5% CO_2_. CHIKV isolate 99659 (GenBank:KJ451624), originally isolated from a 33 year old male in the British Virgin Islands with a single round of amplification on Vero cells, was obtained from Brandy Russell (Centers for Disease Control and Prevention, Ft. Collins, CO, USA). Virus stocks were prepared by inoculation onto a confluent monolayer of C6/36 mosquito cells. This CHIKV strain is related phylogenetically to strains recently identified in Asia with most of them sharing a specific four amino-acid deletion in the nsP3 gene [3], and is representative of CHIKV strains circulating in Colombia [27].

### Mosquito strains and colony maintenance

*Ae*. *aegypti* used in this study were maintained at the University of Wisconsin-Madison as previously described [26]. Two populations of mosquitoes were used in this study. Wild type (WT) mosquitoes (not infected with *Wolbachia*) were established from eggs collected from ovitraps placed around the municipality of Bello, a northwest suburb of Medellin, Colombia. The *Wolbachia*-infected (*w*MelCOL; infected with the *w*Mel strain of *Wolbachia pipientis*) mosquito line was created by crossing uninfected field strains with a *w*Mel-infected laboratory strain of *Ae*. *aegypti* essentially as described in [27]. The *w*Mel-infected laboratory population of *Ae*. *aegypti* originated from eggs provided by Scott O’Neill (Monash University, Victoria Australia). *Wolbachia* infection status was routinely verified using PCR with primers specific to the *IS5* repeat element [19].

### Exposure to infective bloodmeal

Mosquitoes were exposed to CHIKV by feeding on isoflurane anesthetized CHIKV-infected *Ifnar-/-* mice. These mice have abrogated type I interferon signaling and as a result develop lethal infection, with muscle, joint, and skin serving as primary sites of replication [28,29]; as well, as developing high viremia. *Ifnar-/-* mice on the C57BL/6 background were obtained from Eva Harris (University California-Berkeley, Berkeley, CA) and were bred in the pathogen-free animal facilities of the University of Wisconsin-Madison School of Veterinary Medicine. Groups of three and six week-old mixed sex mice were used for mosquito exposures because viremia varied with age. Mice were infected in the left, hind foot pad with either 10^3^ plaque forming units (PFU) of CHIKV in 50 μl of animal diluent (AD: 1% heat-inactivated FBS in Dulbecco’s PBS) for three week-old mice or 10^4.5^ PFU of CHIKV in 50 μl of AD for six week-old mice. Uninfected mosquitoes (both WT and *w*MelCOL) were allowed to feed on mice two days post infection at which time sub-mandibular blood draws were performed and serum was collected to verify viremia. Three week-old mice yielded an infectious bloodmeal concentration of 9.51 log_10_ PFU/ml ± 0.09 (mean ± standard deviation; n = 6) and six week old mice yielded an infectious bloodmeal concentration of 6.90 log_10_ PFU/ml ± 0.14. These bloodmeal titers were consistent with human viremias observed in the field [30–32].

### Vector competence

Infection, dissemination, and transmission rates were determined using long established procedures [33,34]. Briefly, three- to six-day-old female mosquitoes were sucrose starved for 14 to 16 hours prior to exposure to mice. Mosquitoes that fed to repletion were separated into cartons and maintained on 0.3 M sucrose in an environmental chamber at 26.5°C ± 1°C, 75% ± 5% relative humidity, and with a 12 hour photoperiod within the Department of Pathobiological Sciences BSL3 Insectary facility at the University of Wisconsin-Madison. All samples were screened by plaque assay on Vero cells. Dissemination was indicated by virus-positive legs. Transmission was defined as release of infectious virus with salivary secretions, i.e., the potential to infect another host, and was indicated by virus positive-salivary secretions.

### Plaque assay

All CHIKV screens and titrations for virus quantification were completed by plaque assay on Vero cell cultures. Duplicate wells were infected with 0.1 ml aliquots from serial 10-fold dilutions in growth media and virus was adsorbed for one hour. Following incubation, the inoculum was removed, and monolayers were overlaid with 3 ml containing a 1:1 mixture of 1.2% oxoid agar and 2X DMEM (Gibco, Carlsbad, CA) with 10% (vol/vol) FBS and 2% (vol/vol) penicillin/streptomycin. Cells were incubated at 37°C in 5% CO_2_ for two days for plaque development. Cell monolayers then were stained with 3 ml of overlay containing a 1:1 mixture of 1.2% oxoid agar and 2X DMEM with 2% (vol/vol) FBS, 2% (vol/vol) penicillin/streptomycin, and 0.33% neutral red (Gibco). Cells were incubated overnight at 37°C and plaques were counted.

### Statistical analysis

Infection, dissemination, and transmission rates were analyzed using an Exact unconditional test [35]. Saliva CHIKV titers were analyzed using a Bootstrap t-test and survival data were analyzed using Kaplan-Meir analysis and log-rank statistics.

## Results and Discussion

### *Wolbachia* influences the mosquitoes’ permissiveness to CHIKV infection

In Colombia, all four DENV serotypes actively circulate in many parts of the country and there has been a significant increase in the number of severe dengue cases since re-emergence [36]. The rise in cases coincided with an increase in *Ae*. *aegypti* populations that also have expanded into new geographic locales. Similar to the country as a whole, Medellin, the second largest city in the country, also had a significant increase in dengue cases, despite the presence of a national integrated vector control strategy. This provided the impetus for new approaches to preventing DENV transmission. In fact, deployment of *Wolbachia-*infected *Ae*. *aegypti* began in Medellin early last year (2015) to assess the efficacy of this technology in reducing DENV transmission in endemic populations. Not surprisingly, CHIKV reached Colombia in August 2014 [25], and since its introduction, there have been over 300,000 cases of CHIKV detected. Again, current vector control measures were insufficient in preventing invasion of this virus into the country or controlling it after invasion. Although primarily designed as a biocontrol tool for DENV, evidence suggests that *Wolbachia* can limit infection in *Ae*. *aegypti* with CHIKV [23]; therefore, *Wolbachia-*infected *Ae*. *aegypti* could potentially be used to simultaneously control DENV and CHIKV. As a result, we evaluated whether Colombian mosquitoes infected with the *w*Mel strain of *Wolbachia* reduced CHIKV transmission potential.

Here, we verified that the phenotype of reduced vector competence existed in *Wolbachia*-infected laboratory colonies of Colombian *Ae*. *aegypti* for CHIKV. Adult, female, mosquitoes were exposed to infectious bloodmeals containing CHIKV and mosquitoes that ingested blood containing virus were assayed for infection, dissemination, and transmission potential at 7 and 14 days (d) post feeding (PF). As expected, infection, dissemination, and transmission rates were high for WT exposed to blood containing CHIKV at a concentration of 9.51 log_10_ PFU/ml (Table 1). Although viral titer in the bloodmeal was high, CHIKV viremia in humans can vary drastically (ranging from 10^1^−10^9^ PFU/ml), and therefore was consistent with observations in the field [30–32]. Furthermore, evidence suggested that infection and dissemination rates were dose-dependent and rates increase with the titer of the ingested bloodmeal (see [37] for review). Our first goal then was to determine if there was a threshold in which a high viral infectious dose could overwhelm the system and negate the protection conferred by *Wolbachia*. Interestingly, there was a significant reduction (Exact Unconditional Test) in infection, dissemination, and transmission rates for *w*MelCOL exposed to blood containing CHIKV; i.e., *Wolbachia* infection in Colombian *Ae*. *aegypti* completely blocked CHIKV transmission at 7d PF and significantly reduced infection and dissemination rates at 14d PF (Table 1). These data were consistent with other strains of *w*Mel-infected *Ae*. *aegypti* when exposed to CHIKV [23] or DENV [21,38]; i.e., *Wolbachia* infection does not completely ablate transmission of virus, but rather delays the extrinsic incubation period (EIP) of the virus and reduces the transmission potential of CHIKV-infected mosquitoes.

**Table 1:** Vector competence of Colombian mosquitoes following peroral infection.*

CHIKV						
	7d PF			14 d PF		
Mosquito	I	D	T	I	D	T
**WT**	97 (n = 30)	100 (n = 29)	55 (n = 29)	100 (n = 31)	100 (n = 31)	61 (n = 31)
***w*MelCOL**	37 (n = 30)	45 (n = 11)	0	19 (n = 26)	20 (n = 5)	20 (n = 5)
***p* value**^**†**^	0.0001	0.0001	0.0008	0.0001	0.0001	0.0997

*I, % Infected; D, % Disseminated (of infected); T, % Transmitting (of infected); bloodmeal titer = 9.51 log_10_ PFU/ml of CHIKV.

^†^Calculated using an Exact unconditional test

### Dynamics of infective mosquitoes

**Fig 1:**
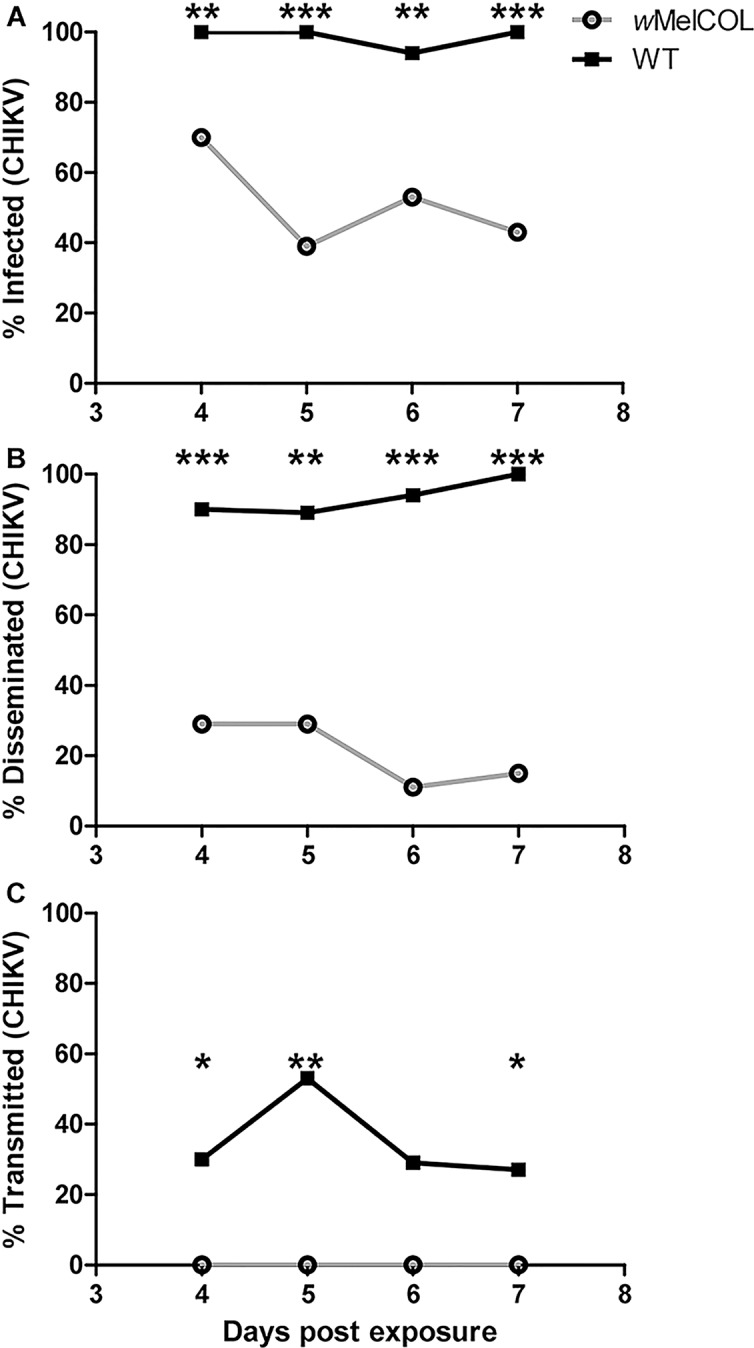
Infection dynamics through time for WT (black line) and *w*MelCOL mosquitoes (gray line) orally infected with 6.90 log_10_ PFU/ml of CHIKV. Mosquitoes were examined at days 4–7 to determine infection, dissemination, and transmission efficiencies. Infection efficiency corresponds to the proportion of mosquitoes with virus-infected bodies among the tested ones. Dissemination efficiency corresponds to the proportion of mosquitoes with virus-infected legs, and transmission efficiency corresponds to the proportion of mosquitoes with infectious saliva among those infected. *, significant reduction in infection rates (*p<0.05, **p<0.01, ***p<0.001). **A).** Percent infected (4d, n = 20 for *w*MelCOL and WT; 5d, n = 18 for *w*MelCOl and n = 19 for WT; 6d, n = 17 for *w*MelCOL and n = 18 for WT; 7d n = 30 for *w*MelCOL and WT. **B).** Percent disseminated (of infected). **C.)** Percent transmitting (of infected).

**Fig 2:**
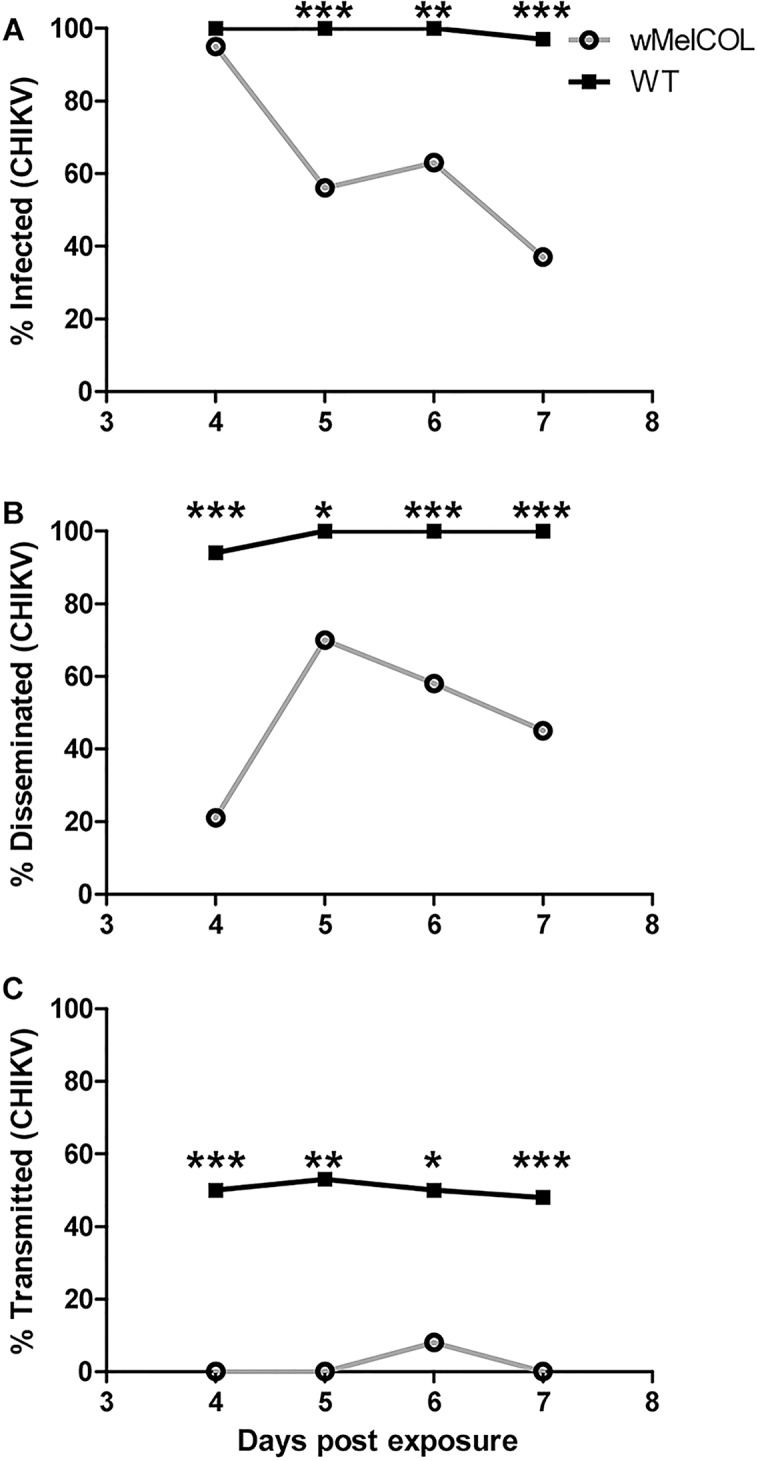
Infection dynamics through time for WT (black line) and wMelCOL mosquitoes (gray line) orally infected with 9.00 log_10_ PFU/ml of CHIKV. Mosquitoes were examined at days 4–7 to determine infection, dissemination, and transmission efficiencies. Infection efficiency corresponds to the proportion of mosquitoes with virus-infected bodies among the tested ones. Dissemination efficiency corresponds to the proportion of mosquitoes with virus-infected legs, and transmission efficiency corresponds to the proportion of mosquitoes with infectious saliva among those infected. *, significant reduction in infection rates (*p<0.05, **p<0.01, ***p<0.001). **A).** Percent infected (4d, n = 20 for *w*MelCOL and n = 18 for WT; 5d, n = 18 for *w*MelCOl and n = 19 for WT; 6d, n = 19 for *w*MelCOL and n = 14 for WT; 7d n = 30 for *w*MelCOL and WT. **B).** Percent disseminated (of infected). **C.)** Percent transmitting (of infected).

**Fig 3:**
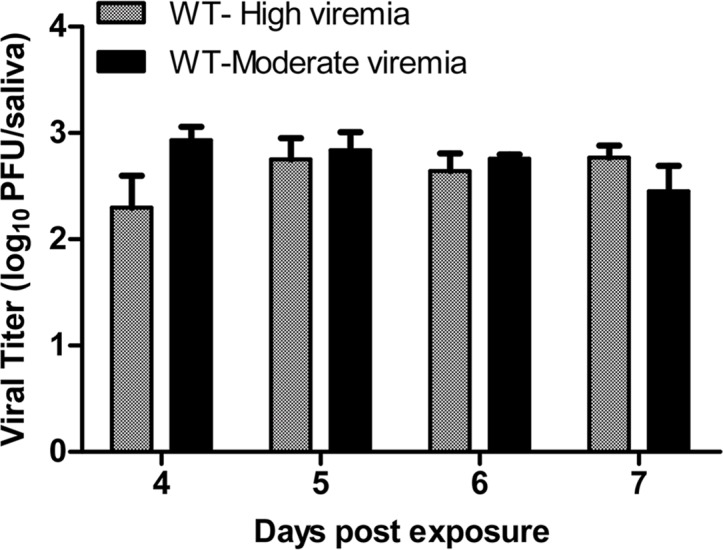
Viral titers in saliva of WT mosquitoes at different days after peroral infection with 6.90 or 9.00 log_10_ PFU/ml of CHIKV. Error bars represent the Bootstrap 95% confidence interval for the mean.

Recently, Ye et al. (2015) demonstrated that *Wolbachia*-infected mosquitoes exhibited fewer infective days compared to WT mosquitoes, and their data suggested that *Wolbachia*-infected mosquitoes were infective at earlier timepoints [38]; however, they relied on qRT-PCR to detect and quantify virus, which does not differentiate infectious from non-infectious virus [39]. The plaque assays used here quantified infectious particles. Furthermore, it also has been demonstrated that this strain of CHIKV could be detected in the saliva of *Ae*. *aegypti* as early as 3d PF, albeit at very low levels [40]. To ascertain if *w*MelCOL had the potential to transmit CHIKV at earlier time points, we assessed the dynamics of infection in WT and *w*MelCOL over time following an infectious bloodmeal more in agreement with viremias detected in Colombian patients (6.90 log_10_ PFU/ml) [25] versus a high viremic infectious bloodmeal (>9.0 log_10_ PFU/ml). After a CHIKV-infected bloodmeal of moderate viremia, WT mosquitoes quickly became infective (Fig 1A–1C) and peaked at 53% infective (10/19) at 5d PF (Fig 1C). In contrast, *w*MelCOL remained non-infective over the duration of seven days (Fig 1C), but a large proportion (39%-70%) of *w*MelCOL had established infections (Fig 1A) and a moderate number (11%-29%) also disseminated virus (Fig 1B). Likewise, after a CHIKV-infected bloodmeal of high viremia, WT mosquitoes quickly became infective (Fig 2A–2C) and maintained infectivity (Fig 2C). In contrast, *w*MelCOL remained non-infective over the duration of seven days (Fig 2C), with the exception of a single mosquito with CHIKV-positive saliva on day six. A large proportion (up to 95% at 4d PF) of *w*MelCOL had established infections (Fig 2A) and a moderate number (21–70%) also disseminated virus (Fig 2B). Infectious virus also was detected in the saliva of *w*MelCOL on day 14 PF (Table 1). WT mosquitoes exposed to a bloodmeal of high viremia had viral titers in the saliva consistent with WT exposed to a bloodmeal of moderate viremia (Fig 3).

### Mosquito survival post chikungunya virus infection

**Fig 4:**
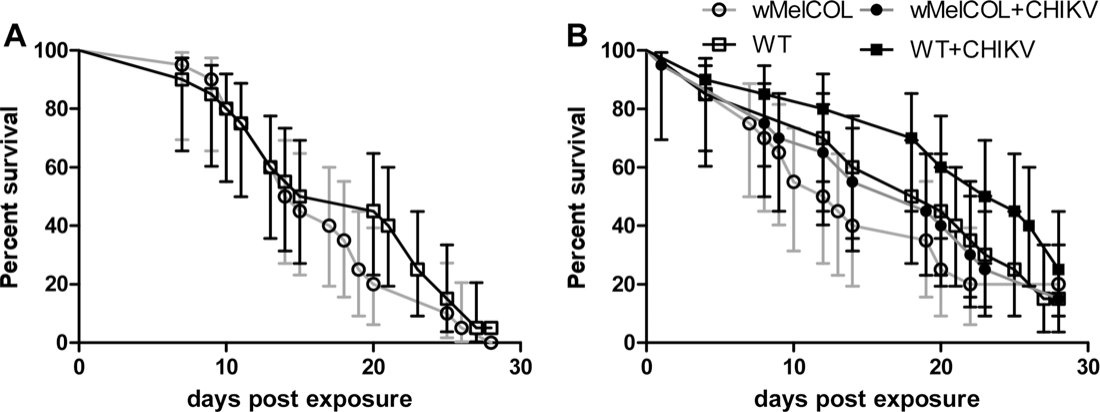
**Survival curves of WT (black line) and *w*MelCOL mosquitoes (gray line) orally infected with 6.90 (A) or 9.00 (B) log**_**10**_ **PFU/ml of CHIKV.** Error bars represent 95% confidence interval.

We then investigated whether CHIKV had a negative effect on mosquito survival, because probability of daily survival is an important parameter in estimating vectorial capacity. It is critically important to understand how virus infection impacts vector survival if accurate predictions of transmission dynamics are to be made, because low mosquito survival will reduce the likelihood of onward transmission of the infecting virus to a new host. There has been inconsistency among reports of the effects of arboviruses on mosquito survival, and to our knowledge no reports on the impact of CHIKV infection on mosquito survival. A recent meta-analysis involving various vector-virus combinations found that, overall, arboviruses do reduce the survival of their mosquito vectors [41]. And, others have suggested that the presence of *w*Mel infection can lengthen the lifespan of mosquitoes as compared to WT following DENV infection, suggesting that DENV infection is costly to mosquitoes and that *Wolbachia* is conferring some protection to the host [38]. Here, the presence of *w*Mel infection had no impact on the lifespan of mosquitoes as compared to WT following CHIKV infection (*p* = 0.369 and *p* = 0.429; Fig 4A and 4B, respectively), nor was there any indication that CHIKV infection was overly costly to WT mosquitoes (Fig 4B). Certainly, mosquitoes survived the relatively short EIP of CHIKV (Figs 1 and 2). It also is important to note that we explored the effects of naturally acquired CHIKV infection (i.e., exposure to virus was accomplished by feeding on a viremic host) on mosquito survival; whereas, most previous studies have relied on animal blood spiked with cultured virus, which may or may not have influenced the magnitude of the observed effect. Furthermore, recent studies suggested that viral titer in the bloodmeal might impact mosquito survival; i.e., high viral titers in the blood lead to increased mosquito mortality [42]. Here, unusually high mortality was not observed in mosquitoes exposed to blood containing CHIKV at a concentration of >9.0 log_10_ PFU/ml, i.e., a very high viral titer in the bloodmeal (Fig 4). These data are in concordance with a recent study by Carrington et al. (2015) that demonstrated that DENV infection adds minimal cost to *Ae*. *aegypti* when mosquitoes were exposed to DENV by feeding on infected humans, and there was no relationship between survival and human plasma viremia levels [43]. Although a direct comparison cannot be made, our data suggest that the relationship between CHIKV and *Ae*. *aegypti* is also relatively benign; but, we cannot rule out that CHIKV and/or *Wolbachia* infection may impart additional costs not measured here, e.g., reduced fecundity [44]. Finally, *Wolbachia* biocontrol depends on *Wolbachia* infections being maintained stably at high levels within natural mosquito populations as well as continuing to exhibit virus interference. *Wolbachia* may not stably persist if there are changes in maternal transmission, cytoplasmic incompatibility, and/or fitness effects to the mosquito as a result of *Wolbachia* infection. *Wolbachia* infection did not shorten the lifespan of infected mosquitoes (Fig 4B), which bodes well for the success of this strategy, but work still is needed to assess the long-term stability of infection and changes in host fitness effects following invasion in Colombia.

In sum, *Wolbachia* biocontrol has been proposed primarily as a tool to control DENV transmission [19], but *Wolbachia* infections also confer protection for *Ae*. *aegypti* against CHIKV and to some extent yellow fever virus (YFV) [23] as well. And, as a result of the explosive outbreak of CHIKV and now Zika virus in the Western hemisphere [12,45–47], all four of these viruses co-circulate in many parts of the tropics. The possibility exists that *Wolbachia* biocontrol could be used as a multivalent strategy for all of these *Ae*. *aegypti*-transmitted arboviruses. At the very least, these results warrant further exploration, both in the laboratory and the field, on the feasibility of expanding this technology beyond DENV and informing whether *Wolbachia* biocontrol can be used to supplement or replace existing vector control strategies.
